# Contributing to Understand the Crosstalk between Brain and Periphery in Methylmercury Intoxication: Neurotoxicity and Extracellular Vesicles

**DOI:** 10.3390/ijms221910855

**Published:** 2021-10-07

**Authors:** Gabriela de Paula Arrifano, Marcus Augusto-Oliveira, Megan Sealey-Bright, Jaezah Zainal, Luciana Imbiriba, Luanna Melo Pereira Fernandes, Cristiane Socorro Ferraz Maia, Daniel Anthony, Maria Elena Crespo-Lopez

**Affiliations:** 1 Laboratório de Farmacologia Molecular, Instituto de Ciências Biológicas, Universidade Federal do Pará, Belém 66075-110, Brazil; arrifanogabriela@gmail.com (G.d.P.A.); marcusoliveira@globo.com (M.A.-O.); imbiriba.luciana@gmail.com (L.I.); 2 Department of Pharmacology, University of Oxford, Oxford OX1 3QT, UK; MBright@rosetreestrust.co.uk (M.S.-B.); jaezah.zainalazman@pharm.ox.ac.uk (J.Z.); 3 Laboratory of Pharmacology of Inflammation and Behavior, Faculty of Pharmacy, Institute of Health Science, Federal University of Pará, Belém 66075-110, Brazil; luannafe@hotmail.com (L.M.P.F.); crismaia@ufpa.br (C.S.F.M.)

**Keywords:** exosomes, mercury, MeHg, exposure, pollution, pollutant, CNS, S100b, real time, qPCR

## Abstract

Human exposure to methylmercury (MeHg) is currently high in regions such as the Amazon. Understanding the molecular changes associated with MeHg-induced neurotoxicity and the crosstalk with the periphery is essential to support early diagnoses. This work aimed to evaluate cellular and molecular changes associated with behavioral alterations in MeHg acute exposure and the possible changes in extracellular vesicles (EVs) number and S100β content. Adults male Wistar rats were orally treated with 5 mg/kg for four days. Behavioral performance, molecular and histological changes in the cerebellum, and plasma EVs were assessed. MeHg-intoxicated animals performed significantly worse in behavioral tests. MeHg increased the number of GFAP+ cells and GFAP and S100β mRNA expression in the cerebellum but no change in NeuN+ or IBA-1+ cells number was detected. The number of exosomes isolated from plasma were decreased by the metal. S100B mRNA was detected in circulating plasma EVs cargo in MeHg exposure. Though preliminary, our results suggest astrocytic reactivity is displaying a protective role once there was no neuronal death. Interestingly, the reduction in exosomes number could be a new mechanism associated with MeHg-induced neurotoxicity and plasma EVs could represent a source of future biomarkers in MeHg intoxication.

## 1. Introduction

Mercury is included in the top ten most dangerous pollutants [1], and while there is a global commitment to reduce its emissions into the environment (Minamata Convention—https://www.mercuryconvention.org/, accessed on 4 October 2021), levels continue to increase [2]. This metal is ubiquitous and once it reaches the watershed it can be biotransformed by methanogenic bacteria into methylmercury (MeHg). In the latter form, it suffers bioaccumulation and biomagnification through the food chain, which represents the main route of human exposure by the ingestion of contaminated fish [2]. Human exposure to MeHg, the most toxic form of mercury, is currently high in regions such as the Amazon [3,4] due to the high consumption of piscivorous fish (with the highest levels of the metal [5] in the diet of traditional populations [6]).

The central nervous system (CNS) is the principal target for MeHg toxicity, which can give rise to psychomotor alterations, damage to the visual system, and tremors, among other neurological symptoms [7,8]. The cerebellum is particularly susceptible to MeHg [9,10]. Indeed, a hallmark of MeHg intoxication is cerebellar ataxia owing to the dysfunction of granule cell neurons [11]. MeHg also affects microglia (the main arm of defense system of the CNS [12]) and astrocytes (which regulate several critical events including synapse formation, plasticity, and elimination ultimately regulating animal behavior [13]) [14,15]. Noteworthy, the precise role performed by astrocytes in MeHg intoxication is currently under intense debate [15].

An accurate diagnosis of mercury intoxication can be very problematic as it is associated with many symptoms [16]. Furthermore, as a consequence of the influence of genetic susceptibility, dose, and time of exposure, it is often difficult to find a direct correlation between mercury levels (i.e., exposure) and symptoms [16,17,18]. Thus, there is an urgent need for the development of peripheral biomarkers to support the early diagnosis of mercury-induced neurotoxicity [19,20]. Recently, both protein and mRNA of S100 calcium-binding protein B (S100β) were proposed as blood biomarker of mercury intoxication [17,18]. The increased release of S100β by glia is a well-documented response to acute and chronic brain pathology [21]. The possibility of measuring peripheral changes of this biomarker, directly associated to its changes in CNS, would allow for earlier diagnosis and intervention.

In this context, there is an urgent need to better understand the crosstalk between the brain and the periphery in MeHg-induced neurotoxicity. In recent years, extracellular vesicles (EVs) have emerged as important elements in cell communication and as potential source of biomarkers for disease [22,23]. EVs are small bi-layer-enclosed vesicles (nano to microsized), which are released for intercellular communication locally and between organs [24,25]. There are three main subtypes of EVs: (1) exosomes, which are the smallest EVs (40–100 nm in diameter); (2) microvesicles (50–1000 nm in diameter); and (3) apoptotic bodies (500–2000 nm in diameter) [24,25]. EVs are known to cross the blood brain barrier [22,25] and to have a role in the crosstalk between brain and periphery. For example, CNS-derived plasma exosomes in living humans have been found to be altered in preclinical Alzheimer disease studies [26,27,28]. As a consequence, changes in the peripheral EVs population also afford a potential biomarker for evaluating MeHg toxicity.

Here we sought to evaluate how the EVs populations is altered and how S100β levels related to neural and behavioral alterations in MeHg acute exposure.

## 2. Results

### 2.1. MeHg Induced Motor Impairment

To test for neural and behavioral changes, rats were first treated with MeHg (5 mg/kg, via oral) for four consecutive days. Behavioral functions such as balance and coordination were tested 24 h after the last treatment. The animals underwent open field and rotarod tests.

**Figure 1:**
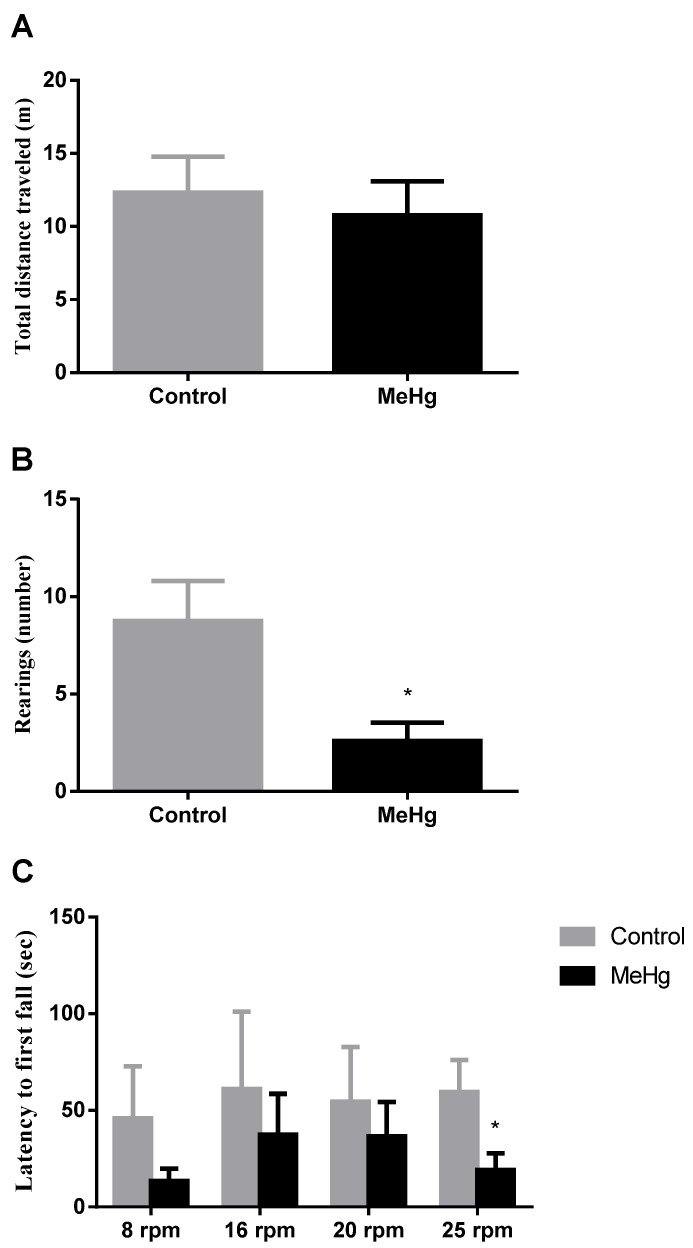
Effects of methylmercury (MeHg) in motor performance. Total distance walked (**A**) and number of rearings (**B**) registered in the open field test, and latency time to the first fall in the rotarod test (**C**) were analyzed in rats orally exposed to water (Control) or MeHg 5 mg/kg (MeHg) for four consecutive days. Behavioral tests were performed 24 h after the last dose of treatment. Results are expressed as mean ± SEM. *n* = 4–5 per group. Student *t* test, * *p* < 0.05 vs. control.

Animals treated with MeHg showed significant decrease in both number of rearings registered in the open field test (Figure 1B), and latency time to the first fall in the latest stage of the rotarod test when given at the highest dose, as compared to control animals (Figure 1C), suggestive of MeHg toxicity.

### 2.2. MeHg Induced Histological Changes

The animals were sacrificed 24 h after the treatment, perfused and the brains were fixed for histology. We wanted to assess whether MeHg toxicity affected the number of cells in the cerebellum as a marker of damage. Immunohistochemistry for neuronal nuclei (NeuN), ionized calcium binding adaptor molecule 1 (IbaI) or glial fibrillary acidic protein (GFAP) was used to visualize neurons, microglia and astrocytes, respectively.

**Figure 2:**
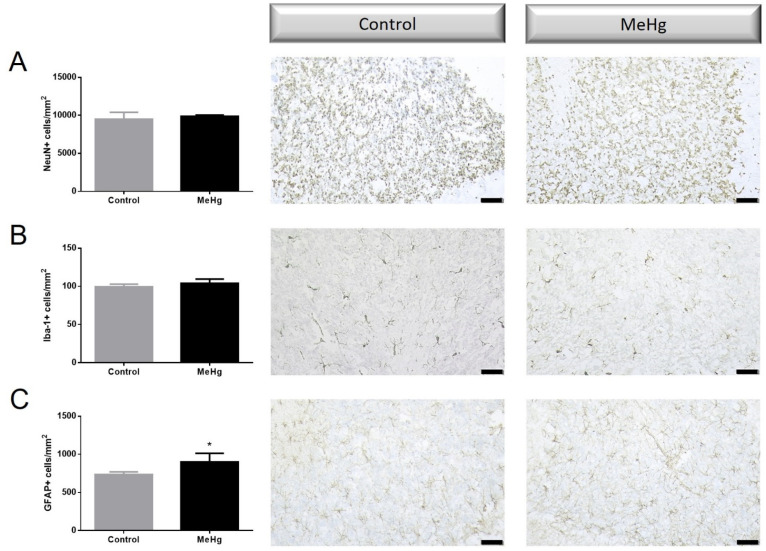
Effects of methylmercury (MeHg) in neurons, microglia and astrocytes in cerebellum. Quantitation of immunostaining cells (graphics on the left) and representative photomicrographs (center and right) of the cerebellum of rats orally exposed to water (Control) or MeHg 5 mg/kg (MeHg) for four consecutive days: (**A**) neurons (positive cells for NeuN marker, NeuN+); (**B**) microglial cells (positive for Iba-1 marker, Iba-1+); and (**C**) astrocytes (positive cells for GFAP marker, GFAP+). Immunohistochemistry was performed 24 h after the last dose of treatment. Results are expressed as mean ± SEM. *n* = 3–4 per group. Student *t* test, * *p* < 0.05 vs. control. Scale bar = 50 µm.

In fact, MeHg treatment appeared to induce a small but significant increase in the number of GFAP+ cells (Figure 2C) but no change in NeuN+ or IbaI+ cells (Figure 2A,B), suggesting astroglial reactivity.

### 2.3. MeHg Induced Changes in Neuronal and Astrocytic Reactivity Molecular Markers

**Figure 3:**
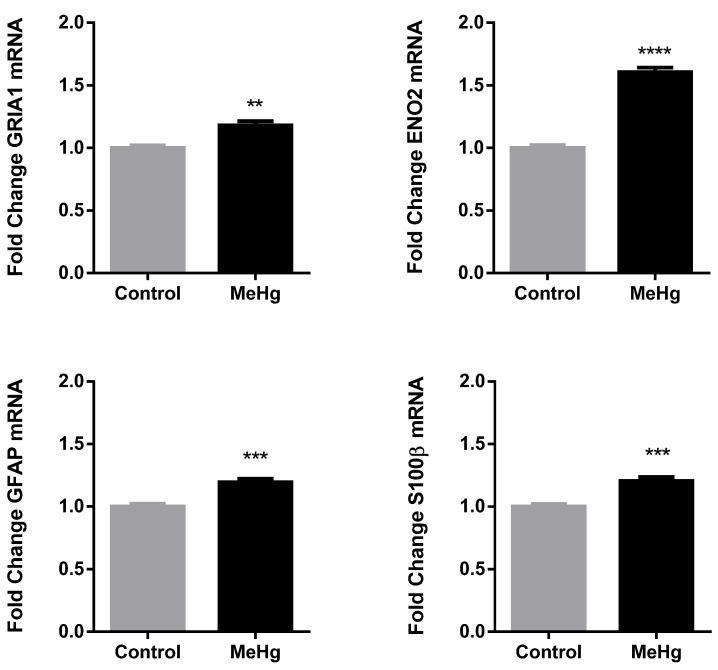
Effects of methylmercury (MeHg) in gene expression in the cerebellum. Expression of GRIA1, ENO2, GFAP and S100β genes in the cerebellum of rats exposed to water (Control) or MeHg 5 mg/kg (MeHg) for four consecutive days were assessed by RT-qPCR 24 h after the last dose of treatment. Results are expressed as mean ± SEM. *n* = 4–5 per group. Student *t* test ** *p* < 0.01; *** *p* < 0.001; **** *p* < 0.0001 vs. control.

To understand the effect that MeHg toxicity has on neuronal and astrocytic cells in the cerebellum, an array of markers was probed using RT-qPCR. The neuronal disturbance was assessed through mRNA expression of GLUT1R subunit of AMPA receptor (GRIA1) and neuronal specific enolase (ENO2) and the astrocytic reactivity was evaluated through mRNA expression of GFAP and S100β. Though preliminary, we found that MeHg treatment induced GRIA1, ENO2, GFAP and S100β gene expression in the cerebellum (Figure 3) suggesting both neuronal injury and astrocyte reactivity at molecular levels.

### 2.4. Plasma Extracellular Vesicles

**Figure 4:**
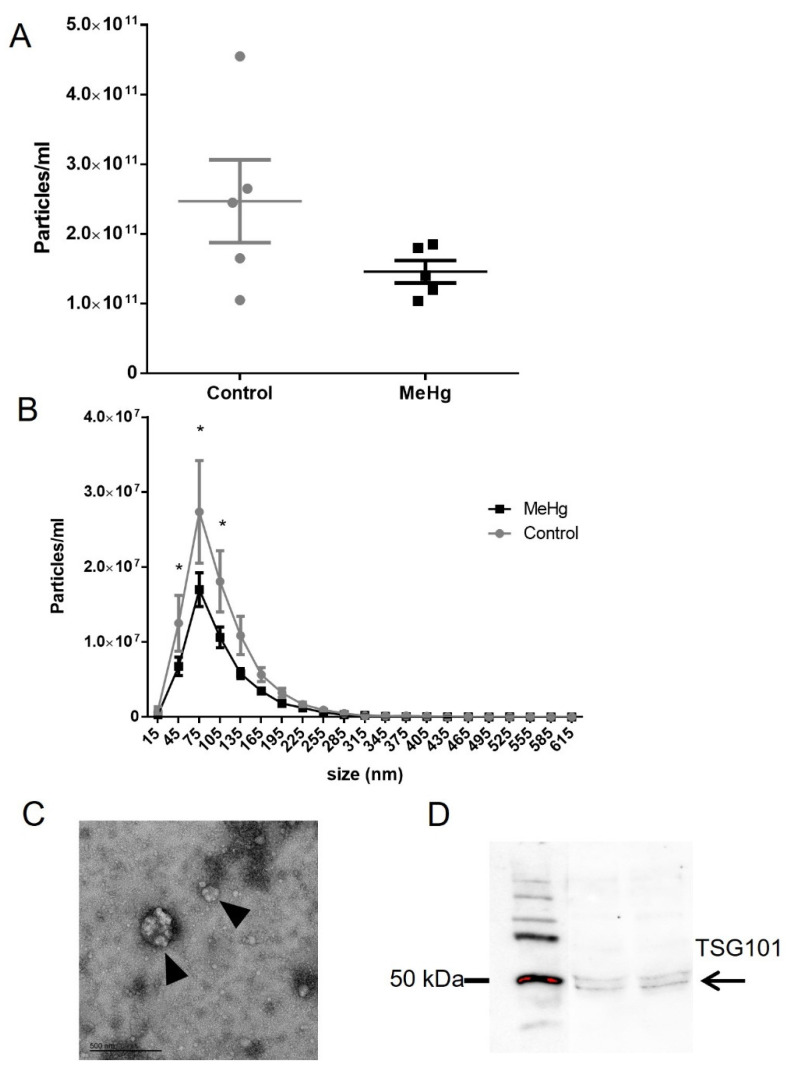
Effects of methylmercury (MeHg) in circulating plasma extracellular vesicles (EVs). Rats were orally exposed to water (Control) or MeHg 5 mg/kg (MeHg) for four consecutive days and EVs were isolated from plasma: (**A**) total number of EVs (each dot representing each treated animal); (**B**) number of smaller EVs (45, 75, and 105 nm); (**C**) representative electron micrograph (scale bar of 500 μm) showing the detected EVs (black arrow head); and (**D**) western blot showing that EVs were immunopositive for the TSG101 (black arrow), a protein marker of EVs. EVs isolation was performed 24 h after the last dose of treatment. Results are expressed as mean ± SEM. *n* = 4–5 per group. Student *t* test (A) and two-way ANOVA (B), * *p* < 0.05 vs. control.

As EVs have been found to contain biomarkers for other conditions, the number of EVs was assessed here using nanoparticle tracking analysis (NTA) and the EVs cargo was determined by RT-qPCR. We found that MeHg treatment induced no change in the overall total number of EVs (Figure 4A) however the treatment did result in significant lower number of smaller EVs (45, 75 and 105 nm) isolated from plasma (Figure 4B).

Images acquired by transmission electron microscopy revealed the presence of extracellular vesicles isolated from plasma (Figure 4C). Western blotting showed that EVs were immunopositive for the tumour susceptibility gene 101 protein (TSG101), a protein marker of EVs (Figure 4D).

**Figure 5:**
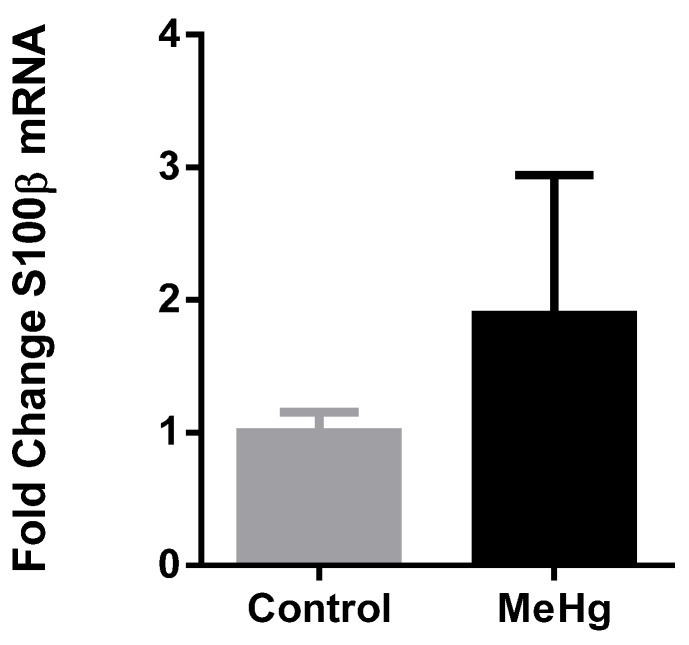
S100β mRNA in circulating plasma EVs cargo. Rats exposed to MeHg 5 mg/kg for four consecutive days presented no significant change in S100β mRNA in EVs cargo. Results are expressed as mean ± SEM. *n* = 4–5 per group. Student *t* test *p* > 0.05.

Next, in order to investigate whether circulating plasma EVs carried S100β mRNA, we extracted RNA from the EVs fraction and probed to S100β using RT-qPCR. Thus, in this preliminary approach, we detected the S100β mRNA inside of the EVs, although the possible difference between groups (MeHg vs. control) did not reach statistical significance (Figure 5).

## 3. Discussion

In the present manuscript we assessed molecular and histological changes in the cerebellum in an acute MeHg-intoxication model in rats. This is the first paper which attempts to understand the crosstalk between the brain and periphery in a model of MeHg brain injury.

An acute exposure model by oral gavage was used to test the methylmercury-induced neurotoxicity. High levels of human exposure are usually found in regions such as the Amazon, mainly due to the consumption of contaminated fish [2,5]. The model used in our work, to simulate the most common pathway (oral) of human exposure to MeHg, has previously been used to show gastrointestinal changes in aquaporines’ gene expression, as a new mechanism of absorption of MeHg in the gut [29]. Thus, this dose is able to produce significant changes at molecular level, allowing the understanding of early stage of MeHg intoxication. Here we showed that MeHg exposure produced motor impairment once animals treated with the metal performed significantly worse in vertical exploitation on the open field as well as in the last stage of rotarod test (Figure 1). Such reduced vertical exploration has previously been observed even with MeHg subtoxic-dose exposure [30]. However, although an open field test has already been used to evaluate motor activity in mercury intoxication, previous data showed that it may not be the most sensitive test, probably since the performance is mainly related to the motor cortex [31,32]. Rotarod is well-documented to be better at evaluating neurobehavioral alterations caused by MeHg [31,33,34,35]. Indeed, although we found no change in the total distance travelled in the open field test, we did find a significantly decreased latency to the first fall in the rotarod test at 25 rpm. These results strongly suggest that the brain injury is related to damage in the cerebellum.

It has been reported that the cerebellar region is severely injured in rat models of MeHg intoxication [36,37]. Our results demonstrated that MeHg increased the number of GFAP+ cells (Figure 2) as well as mRNA expression of GFAP and S100β (Figure 3) in the cerebellum, pointing to astrocytic reactivity. Moreover, neuronal disturbance was detected by the increased mRNA expression of GRIA1 and ENO2 in this brain area (Figure 3). However, MeHg induced no significant changes in number of neurons (NeuN+ cells) or microglia (IBA-1+ cells) in the cerebellum (Figure 2), in our model, suggesting that the increase in ENO2 (neuronal injury) and GRIA1 (indirectly indicative of glutamatergic imbalance) mRNA would not be sufficient to culminate in excitogenic cell death only 24 h after last dose of treatment. However, this acute exposure was enough to lead to astrocytic reactivity, as shown by the significant increase in number of GFAP positive cells (Figure 2) and increased expression of GFAP and S100β mRNA (Figure 3).

Our data reinforces the involvement of glutamatergic toxicity, a well-documented mechanism of MeHg-induced neurotoxicity [15,34,38]. MeHg enhances spontaneous glutamate release from neurons and specifically inhibits the astrocytic glutamate uptake resulting in excessive concentrations of glutamate in the synaptic cleft [15,39]. Previously, the mercury-induced glutamatergic excitotoxicity was shown to be mediated through N-methyl-D-Aspartate receptors (NMDA) in vitro [40]. However, functionally, the most important glutamate receptors to mediate fast excitatory transmission are AMPA receptors (AMPARs) [41]. The increased expression of GRIA1 mRNA in the cerebellum observed in our results (Figure 3), supports the glutamatergic imbalance caused by MeHg, as it would be expected. The long-term consequences of this increased expression of GRIA1 mRNA would eventually include the increased number and, thus, dysregulation of AMPARs with implications in several pathological states and mental health [42]. Recently, a role for MeHg-AMPA receptors interaction was suggested to be involved in amyotrophic lateral sclerosis [43,44]. Moreover, S100β release is increased upon exposure of astrocytes to glutamate [45], also supporting the hypothesis that astrocytic damage is related to an excess of glutamate in the synaptic cleft.

After acute brain injury, ENO2 gene expression was increased in the brain [46]. ENO2 is a glycolytic isoenzyme expressed exclusively in neurons, and some studies have raised the possibility that this enzyme could be associated with neuronal damage (reviewed by [47,48]). Our data showed a significant increased expression of ENO2 in the cerebellum possibly indicating neuronal injury (Figure 3).

Furthermore, our results have additional importance in the translational context. Increased levels of GRIA1 and ENO2 proteins have already been detected in the blood of children exposed to elemental mercury, supporting their value as diagnostic markers [17]. However, this is the first time that both markers have been shown to be altered in brain under MeHg-neurotoxicity.

Following neuronal disfunction, a compensatory response is exerted by astrocytes to protect neurons against death [49]. Astrogliosis is a process believed to have several functions in the brain. Depending on the scenario, astrocytic reactivity could be neuroprotective, leading to repair and return to homeostasis, or detrimental, relating to the inhibition of neuronal axon outgrowth, for instance [50]. Whether astrocytes display a beneficial role or a detrimental one under MeHg-induced neurotoxicity remains controversial (reviewed by [15]). The astrogliosis detected in the present work was assessed using GFAP and S100β (Figure 3), considered the best markers (sensitive and reliable) for detecting astrocytic reactivity following CNS injury [51,52,53]. It is worth noting that the mRNA expression of these markers has been shown to be even more useful for diagnosis than the proteins due to easier conservation, especially for isolated/remote populations [18]. Circulating S100β mRNA has been recently showed to be associated to MeHg exposure in humans [18]. Another interesting fact with valuable meaning in the translational context is that significant S100β mRNA expression was associated with behavioral changes, but it was detected before any significant neuronal death. This supports the use of S100β as a marker for early diagnosis to prevent the worst consequences of MeHg intoxication.

Considering neuronal death is secondary to disturbances in astrocytes [54], and the animals treated with MeHg did not present significant neuronal death (no change in NeuN+ cells number), our data suggest that astrocytes are playing a neuroprotective role in this window of mercury exposure, as recently hypothesized [15]. A growing body of evidence has shown that astrocytes can protect neurons against MeHg-induced neurotoxicity from several pathways (reviewed by [15]). It is hypothesized that in the early stages of MeHg exposure, astrocytes, along with other glial cells, perform cellular and molecular responses to restrict CNS injury. However, in later stages of MeHg exposure, once astrocytes lose their functions or gain maladaptive ones, neuronal damage caused by MeHg would be exacerbated [15].

In CNS injury, signals from the brain are sent to the periphery thorough several mechanism, particularly EVs, which are considered a mode of intercellular communication [22,23,25]. Following brain acute trauma, the circulating number of EVs in the plasma increases and the EVs shed from brain cells are able to incite sick behavior in naïve mice [55,56]. As far as we know, there is no data on how MeHg affects EV homeostasis. In this sense, our seminal work shows that acute exposure to MeHg induced no change in the total number of particles per ml after 24 h of last dose administered (Figure 4A). However, a significant decrease in the number of exosomes (45–105 nm) (Figure 4B) can be already detected at this exposure time window. It is possible that this reduction in exosomes is a novel mechanism of MeHg- induced neurotoxicity, since in the CNS exosomes have been found to play a role in tissue repair and regeneration (reviewed by [57]).

Considering EVs cargo is a potential source of biomarkers [25,26,27,28] and S100β mRNA was recently shown as a strong candidate biomarker of mercury-related neurotoxicity in humans [18], we investigated whether this marker is contained in EVs cargo isolated from plasma. In this work we demonstrated for the first time the presence of S100β mRNA in EVs plasma cargo (Figure 5). This preliminary finding is even more relevant for future clinical applications if we consider the previous work which has shown S100β protein in CSF EV fractions isolated from patients with cognitive impairment [58]. Although the possible difference due to MeHg intoxication in S100β mRNA in EVs plasma cargo did not reach statistical significance, looking at Figure 5, it would be premature to assume that MeHg does not have any influence. Although the increased expression of S100β mRNA would be in line with what we know (previous findings published so far), future studies including a higher sample size are necessary to clarify our results, since we here demonstrated that it is possible to detect S100 β mRNA in circulating plasma EVs, a known biomarker of CNS early injury. This would corroborate previous data showing increased levels of S100β protein in cerebrospinal fluid and serum after brain damage [59,60,61] and mercury exposure [17,18,62], even before the onset of clinical symptoms [17]. The EV content in MeHg exposure scenario could be used as a circulating library for the detection of CNS damage, and this work was the first to approach this idea. Moreover, the RNA contained in EVs is protected against RNase degradation and it is stable under a range of temperature and pH conditions, even with prolonged storage and multiple freeze-thaws [63], making it an excellent alternative for detecting neurotoxicity in isolated/remote populations or locations with limited infrastructure. Currently, South America and Sub-Saharan Africa are responsible for more than one third of the global emissions of mercury, and populations in these regions are among the most exposed populations in the world (UNEP), reinforcing the importance of the research for the development of peripheral biomarkers for early diagnosis of mercury-induced neurotoxicity that are adequate for the study of these populations [20]

## 4. Materials and Methods

### 4.1. Animals Treatment

Twenty-eight male adult Wistar rats (11 weeks old, 220–290 g) were maintained with controlled light and temperature (22 ± 2 °C; 12 h light/dark cycle) with food and water ad libitum. The animals were allowed to acclimatize for one week and were properly manipulated to reduce stress, and all experimental procedures were carried out in accordance with the NIH Guide for the Care and Use of Laboratory Animals after approved by the Committee for Ethics in Experimental Research with Animals of the Universidade Federal do Pará (CEUA 8343070518).

**Figure 6:**
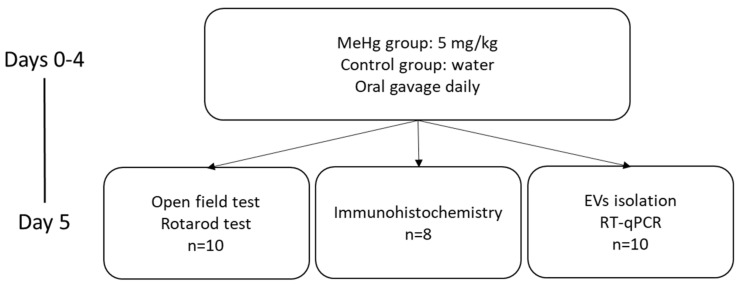
Experimental design.

Animals were randomly allocated into two groups (Control and MeHg) and were treated by oral gastric gavage once a day with water or methylmercury chloride (diluted in water, final dose of 5 mg/kg per body weight)(Figure 6) [29].

### 4.2. Samples Collection

Twenty-four hours after the last treatment, the animals were anesthetized with 40 mg/kg of sodium thiopental, and sample collection was performed.

A set of animals (eight animals, four per group) were transcardially perfused with ice-cold saline containing heparin (20 µL/100 mL), followed by 4% paraformaldehyde (PFA) in 0.1 M phosphate-buffered saline (PBS) (Bento-Torres et al., 2017). Brains were rapidly extracted and post-fixed in 4% PFA, followed by cryoprotection in a 30% sucrose solution, embedded in OCT (Thermo Fisher Scientific, Abingdon, UK) and frozen at −80 °C until use [56].

A different set of animals (10 animals, 5 per group) was used for blood and cerebella collection. Once anesthetized, blood samples were collected via cardiac puncture in EDTA tubes and cerebella were immediately dissected, being snap-frozen in dry ice and stored in −80 °C for posterior analysis.

### 4.3. Behavioral Tests

Twenty-four hours after the last intoxication, a set of animals (10 animals, 5 per group) was conducted to the room experiment and maintained for 1 h of acclimatization with light (12 lux) and noise control. All behavioral tests were carried out from 8:00 a.m. until 18:00 p.m. to avoid circadian cycle alteration.

#### 4.3.1. Open Field Test

Open field test consisted of placing the animal on the center of an acrylic black arena (100 × 100 × 45 cm) and filmed for 5 min. Both vertical (rearing) and horizontal (distance travelled) spontaneous ambulation of the animal were recorded [64]. Distance travelled by the animals was analyzed by the ANY-mazeTM (Stoelting, Wood Dale, IL, USA) software. The rearing was manually registered and the researcher was blinded to treatment group while assessing it.

#### 4.3.2. Rotarod Test

After the open field test, animals were submitted to rotarod test. The equipment consists of an elevated rotation rod motor controlled (Insight, Ribeirão Preto, Brazil), which allows for the rotation velocity to be increased, ranging from 8 rotations per minute (rpm) to 25 rpm, to intensify the difficulty of the test. Briefly, animals were placed on the rotating bar immediately after the velocity of the trial was reached. The trials of the test were at 8, 16, 20, and 25 rpm, with an intertrial interval of 60 s. The cut-off point for each trial was 180 s. The latency of the first fall at each session was recorded by a researcher blinded to treatment group (adapted from [30]).

### 4.4. Platelet-Free Plasma Obtention

Blood samples in EDTA tubes were sat for 30 min at room temperature. After that, samples went through two subsequent centrifugations steps of 2500 g for 15 min at room temperature [65] using a clean plastic tube for the second centrifugation step. Samples were aliquoted and stored in −80 °C for subsequent analysis.

### 4.5. Immunohistochemistry

From fixed brains embedded in OCT, 10 µm thick sections were cut using a cryostat at 20 °C (Leica Microsystems, Milton Keynes, UK). Sagital serial sections were collected with 0.1 M phosphate buffer on gelatinised slides, tissue was allowed to dry overnight and stored at −20 °C. Immunohistochemistry was performed using the avidin-biotin-peroxidase method in a Shandon Sequenza^®^ staining system (Thermo Fisher Scientific, Abingdon, UK). Alternate serial sections were immunolabeled with a polyclonal antibody against neuronal nucleus to detect adult neurons (anti-NeuN, 1:2000, Abcam, Cambridge, UK), Iba-1 to detect microglia/ macrophages (anti-Iba-1; 1:2000, Abcam, Cambridge, UK) and glial fibrillary acidic protein (GFAP) to detect reactive astrocyte (anti-GFAP; 1:700, Dako, Cambridge, UK). All slides were counterstained in a 0.5% cresyl violet acetate solution (pH 3.3). Slides were dehydrated by serial increasing concentrations of alcohol, cleared in xylene and mounted on coverslips with DPX (Fisher Scientific, Loughborough, UK). All slides were randomised and blinded until analysis was complete. Images were taken of the cerebellum with Basler Microscope Software using a light microscope (Leitz Dialux 20) coupled with Microscope Camera 2.0 MP (Basler, High Wycombe, UK). The number of positive cells for each marker was determined using ImageJ (Software version 1.52p, Bethesda, MD, USA).

### 4.6. Relative Quantification of Gene Expression in Brain Areas

Total RNA was extracted from the cerebellum using the QIAshredder and RNeasy Mini Kit (Qiagen, Manchester, UK) as per manufacturer’s instructions. Eluted RNA was quantified using a NanoDrop 1000 spectrophotometer (Thermo Fisher Scientific, Abingdon, UK), RNA was considered acceptable for cDNA conversion if 260/280 and 260/230 ratios were >2. One thousand ng whole RNA was converted to cDNA using the High-Capacity cDNA Reverse Transcription Kit (Applied Biosystems, Abingdon, UK), as per manufacturer’s instructions.

Transcriptase reverse quantitative real time PCR (RT-qPCR) was performed using SYBR Green Mastermix. The validated assay primer mix were purchased from Bio-Rad (Hemel Hempstead, UK). The Unique Assay ID of Bio-Rad primers mix assays were: S100β (qRnoCED0002640); ENO2 (qRnoCED0001449); GRIA1 (qRnoCID0008894); GFAP (qRnoCED0005713).

The reactions contained 25 ng of cDNA, 5 µL of Precision 2X qPCR Mastermix SYBR green (PrimerDesign Ltd., Southampton, UK), 0.5 µL of primer mix assay and nuclease free water to a complete 10 µL final volume. All reactions were made in duplicates and a no template control were run in the same plate in the LightClycler^®^ 480 (Roche Diagnostics, Burgess Hill, UK). The conditions of the running were as follows; enzyme activation at 95 °C for 10 min, 40x denaturation at 95 °C for 15 s and data collection at 60 °C for 1 min, followed by melt curve. The relative quantification was calculated by the ΔΔCt method and expressed as fold-change [66]. GAPDH expression and the control group were used for normalization as the housekeeping gene and the calibrator, respectively.

### 4.7. EV Isolation:

EVs fractions were isolated from platelet-free plasma by differential centrifugation. Briefly, plasma was diluted in PBS (Gibco, Abingdon, UK) and ultracentrifugation at 120,000× *g* for 120 min at 4 °C was used to pellet the EVs using the Beckman Coulter (Brea, CA, USA) Type 70 Ti rotor. The pellet was then resuspended in PBS. The EV pellets were stored at −80 °C until further use.

### 4.8. Nanoparticle Tracking Analysis (NTA):

EV size and quantity were determined using ZetaView Nanoparticle Tracker (Particle Metrix, Cambridge, UK) with the ZetaView software (8.04.02 SP2). The calibration of the instrument was performed using a nanosphere size standard (100 nm diameter; Thermo Fisher Scientific, Abingdon, UK). Instrument pre-acquisition parameters were set to a temperature of 23 °C, sensitivity of 80, frame rate of 30 frames per second (fps), shutter speed of 100, and a laser pulse duration equal to that of shutter duration. Post-acquisition parameters were set to a minimum brightness of 25, maximum size of 1000 pixels, and a minimum size of 5 pixels. Each sample was diluted 1:3000 and 1 mL of diluted EVs was injected into the sample-carrier cell and the particle count was measured at eleven positions, with two cycles of reading per position. Samples were read in duplicate. The cell was washed with PBS (Gibco, Abingdon, UK) after every sample. The mean size and concentration of EVs/mL (±SEM) was calculated for each group.

### 4.9. Transmission Electron Microscopy (TEM)

TEM imaging was performed to validate the presence of EVs in pellets EVs isolated from platelet-free plasma. The pellet of EVs was resuspended in PBS and diluted 1:10 in PBS for optimum imaging, applied freshly in discharged carbon Formvar 3 mm 200 Mesh Cu Grids (AgarScientific, Stansted, UK) for 2 min before being blotted with filter paper. Grids were stained with 2% uranyl acetate for 10 s, blotted and air dried. Grids were imaged in a FEI Tecnai 12 TEM at 12 kV (GAtan OneView CMOS camera, Pleasanton, CA, USA).

### 4.10. Western Blotting

Western blotting was used to confirm the presence of EVs in our samples. Briefly, proteins were resolved by 8–16% Precise Protein Gel SDS–polyacrylamide (Thermo Fisher Scientific, Abingdon, UK) electrophoresis and transferred using a semi-dry system to polyvinylidene difluoride membranes (PVDF) (Bio-Rad, Hemel Hempstead, UK). After blocking, blots were incubated overnight with the primary polyclonal antibody TSG101 (1:250; 5% bovine serum albumin, 4A10 Ab83 Abcam, Cambridge, UK) (marker of presence of EVs). Detection was performed with the appropriate IgG horseradish peroxidase–linked secondary antibody (1:5000; Cell Signaling Technology Danvers, MA, USA). Image analysis was performed using a G:BOX imaging system (Syngene, Cambridge, UK).

### 4.11. Relative Quantification of Gene Expression in Plasma EVs

Total RNA was extracted from EVs fractions using the miRNeasy Mini Kit (Qiagen, Abingdon, UK) as per manufacturer’s instructions. Eluted RNA was checked for quantity and quality using a NanoDrop 1000 spectrophotometer (Thermo Fisher Scientific, Abingdon, UK). Samples with 260/280 and 260/230 ratios > 2 were converted to cDNA using the High-Capacity cDNA Reverse Transcription Kit (Applied Biosystems, Abingdon, UK), as per manufacturer’s instructions. RT-qPCR was performed was described in 4.6. for S100β (qRnoCED0002640) (Bio-Rad, Hemel Hempstead, UK).

### 4.12. Statistical Analysis

Data was analyzed using either unpaired Student-*t* tests or two-way ANOVA to compare groups in Graphpad Prism 6 software (San Diego, CA, USA). The data has been presented as mean ± SEM. *p*-value was set at <0.05 for all analyses.

## Data Availability

All data of this study can be requested to the corresponding author M.E.C.-L. (ecrespo@ufpa.br or maria.elena.crespo.lopez@gmail.com).
